# Brain Exercising Games With Consumer-Grade Single-Channel Electroencephalogram Neurofeedback: Pre-Post Intervention Study

**DOI:** 10.2196/26872

**Published:** 2021-06-15

**Authors:** Pasin Israsena, Suwicha Jirayucharoensak, Solaphat Hemrungrojn, Setha Pan-Ngum

**Affiliations:** 1 National Electronics and Computer Technology Center National Science and Technology Development Agency Pathumthani Thailand; 2 Department of Psychiatry Faculty of Medicine Chulalongkorn University Bangkok Thailand; 3 Cognitive Fitness Research Center Chulalongkorn University Bangkok Thailand; 4 Department of Computer Engineering Faculty of Engineering Chulalongkorn University Bangkok Thailand

**Keywords:** neurofeedback, serious gaming, serious game, brain exercise, cognition training, EEG, aging, cognition, cognitive, brain game

## Abstract

**Background:**

The aging population is one of the major challenges affecting societies worldwide. As the proportion of older people grows dramatically, so does the number of age-related illnesses such as dementia-related illnesses. Preventive care should be emphasized as an effective tool to combat and manage this situation.

**Objective:**

The aim of this pilot project was to study the benefits of using neurofeedback-based brain training games for enhancing cognitive performance in the elderly population. In particular, aiming for practicality, the training games were designed to operate with a low-cost consumer-grade single-channel electroencephalogram (EEG) headset that should make the service scalable and more accessible for wider adoption such as for home use.

**Methods:**

Our training system, which consisted of five brain exercise games using neurofeedback, was serviced at 5 hospitals in Thailand. Participants were screened for cognitive levels using the Thai Mental State Examination and Montreal Cognitive Assessment. Those who passed the criteria were further assessed with the Cambridge Neuropsychological Test Automated Battery (CANTAB) computerized cognitive assessment battery. The physiological state of the brain was also assessed using 16-channel EEG. After 20 sessions of training, cognitive performance and EEG were assessed again to compare pretraining and posttraining results.

**Results:**

Thirty-five participants completed the training. CANTAB results showed positive and significant effects in the visual memory (delayed matching to sample [percent correct] *P*=.04), attention (median latency *P*=.009), and visual recognition (spatial working memory [between errors] *P*=.03) domains. EEG also showed improvement in upper alpha activity in a resting state (open-eyed) measured from the occipital area (*P*=.04), which similarly indicated improvement in the cognitive domain (attention).

**Conclusions:**

Outcomes of this study show the potential use of practical neurofeedback-based training games for brain exercise to enhance cognitive performance in the elderly population.

## Introduction

### Background

The global population aged above 60 years is expected to increase from 900 million in 2015 to 1.4 billion by 2030 and to 2.1 billion by 2050 [1]. Approximately two-thirds of these elderly people live in low-income and middle-income countries [2]. This growing number of elderly will lead to an increase in the incidence of aging-related disorders, including cognitive-related diseases such as dementia, Alzheimer disease, and mild cognitive impairment (MCI), which are increasingly becoming more common [3]. Dementia, the most frequent brain disorder, is differentiated according to the etiology of either vascular dementia or neurodegenerative dementia (such as Alzheimer disease) [4,5]. Dementia is characterized by a devastating reduction in cognitive abilities, including control of behavior, learning, memory, attention/sleep, language, intelligence, perception, as well as functional independence and social relationships. More than 47 million people are living with dementia [6], approximately 60% of whom live in low- and middle-income countries. Driven by population aging, this number is expected to triple by 2050, as the incidence of dementia rises sharply at ages above 75 years. The economic impact has been estimated at US $600 billion per year worldwide [7], including a significant burden of unpaid family caregiving.

In contrast to Alzheimer disease and dementia, which are conditions that are diagnosed at a state that is difficult to treat, MCI—representing an intermediate state between cognitive decline associated with normal aging and dementia [8,9]—is more suitable for targeted preventive measures along with active prevention in the healthy elderly population, as an elderly individual with MCI still has well-preserved functional abilities [10,11]. The global prevalence of MCI in the population aged above 60 years reaches up to 38.60% [12]. MCI is a risk factor for dementia [13] and is associated with a 6-fold increased risk of Alzheimer disease [14]. Indeed, as pharmaceutical-based treatments have yet to be very successful in a late-stage condition such as Alzheimer disease, and may come with certain side effects [15-18], researchers have been exploring the usefulness of alternative strategies to prevent, prolong decline, or even enhance cognitive performance with different methods such as cognitive training [19-22] or neurofeedback training (NFT) [23-25]. Because of the aging trend in developing countries mentioned above, in addition to their demonstrated effectiveness, these methods should be low cost so that they can be beneficial to the majority of the target group.

### NFT as a Solution for Cognitive Decline

NFT is a noninvasive technique to alter brain activity [23-25]. NFT does not actively interfere with the brain but is rather a process during which subjects learn to influence their brain wave pattern by receiving feedback of their brain activity (visual, audio, or other modalities). The controllability of brain rhythms from users often also elicits neural plasticity in the brain that can affect their behaviors and cognitive functions. The use of NFT in medical and therapeutic contexts has continuously attracted interest in research and practice [23]. In particular, in the field that addresses mental problems such as attention deficit hyperactivity disorder (ADHD) or depression [26-31], NFT has been considered a potentially viable tool.

There are several types of NFT based on different brain imaging technologies such hemoencephalographic, low-resolution electromagnetic tomography, functional magnetic resonance imaging, or functional near-infrared spectroscopy. However, the most common type of NFT usually provides brain state information to the user via electroencephalogram (EEG) signals (also called EEG biofeedback). Recorded at the scalp, the EEG is produced by synchronous postsynaptic potentials from thousands to millions of neurons. When amplified, digitized, and plotted, the raw EEG signal appears as a complex oscillatory pattern. Raw EEG signals are observed via frequency bands of interest (ie, delta, 1-4 Hz; theta, 4-8 Hz; alpha, 8-12 Hz; beta, 12-32 Hz; and gamma, 32-60 Hz), usually in the forms of their amplitudes or power spectra.

Brain wave characteristics have been shown to change dramatically depending on the task at hand. Among these, alpha is a particularly interesting oscillation, which is the predominant rhythm in the human brain in a resting state, especially when the eyes are closed [32]. Originally, alpha NFT was considered as simple relaxation training. However, renewed interest has arisen to clarify the relation between the alpha frequency band and cognitive performance [33-35] via indicators such as alpha peak frequency [36,37] and individual upper alpha amplitude/power [38-40]. In resting-state recordings, the alpha peak is clearly visible between 7.5 and 12.5 Hz. Higher alpha peak frequencies (eg, 12 Hz in comparison to 10 Hz) have been shown to correlate positively with high memory performance [36,37] and IQ [41]. Moreover, in terms of alpha amplitude or power, a positive correlation between individual upper alpha amplitude and IQ has been reported (eg, [42,43]). Additionally, as suggested by Klimesch [38], high alpha power during a resting state and low alpha power during the execution of a task were associated with good performance in semantic long-term memory tasks. Despite these promising results, NFT has not yet been translated into mainstream practice. This could be due to limitations of study designs such as lack of a methodological design, therapeutic effect, and validity, as reviewed previously [44,45].

### Consumer-Grade EEG

Until recently, most EEG equipment has been designed for either research or medical purposes. These devices were rather expensive and not easy to operate. An EEG headset usually has multichannel EEG wet sensors held together with a head cap in predefined positions according to the international 10-20 system [46]. Therefore, considerable time is needed for each setup before use and for subsequent cleanup, as these wet sensors needed to be lubricated before each use. The emergence of low-cost consumer-grade EEG headsets offers an easy-to-administer and practical solution via the use of dry or semidry electrodes, along with wireless connectivity, thereby opening the technology for mass use. More importantly, most of the consumer-grade EEGs also have an option for continuous raw EEG reading, as opposed to the batch records usually offered in medical-grade EEGs. This online EEG reading feature is a necessity for systems such as NFT.

There are several consumer-grade EEGs currently available on the market, which differ in terms of the numbers and types of electrodes, positioning, and design. Prominent examples include the Neurosky, Emotiv, or Muse systems. Researchers have been evaluating and comparing these devices with research- or medical-grade solutions [47-52]. In general, the research- or clinical-grade EEGs are more accurate as they employ multichannel wet sensors. For consumer-grade EEG, studies have confirmed that useful EEG readings are possible. These devices are easy to use, but involve dry electrodes with no impedance matching, making them more susceptible to artifacts. They also generally come with a lower number of channels.

Consumer-grade EEG-based systems for applications such as NFT for children with ADHD have already been discussed (eg, [29,30]). We have also published primary research results of NFT using Emotiv [53]. Experience from the trial indicated that although the multichannel Emotiv system provided good results, it was quite time-consuming to administer, as saline still needed to be applied for each sensor. The headset design was also found to be rather bulky for long-term serious use, especially if it was to be shared as part of a multiuser training station.

Alternatively, a single-channel device would provide an even cheaper solution that could expand deployment sites. In particular, the Neurosky design appeared to offer a solid headset design with a single dry electrode placed on the forehead. Although Maskeliunas et al [51] compared the Neruosky device with the multichannel Emotiv device and found the reading quality to be somewhat inferior, it was hypothesized that for NFT application, Neurosky could still be sufficient, especially as the position of the sensor is outside the frontal lobe area, where the cognitive state of interest such as attention occurs. Indeed, in contrast to the results of Maskeliunas et al [51], some authors have gone as far as stating that a single-channel device was preferred [45]. This was supported by the finding of a negative correlation between the number of bands used to compose the feedback signal and the success of training; that is, more complicated protocols that promoted or inhibited several bands worsened the final results.

The aim of this study was therefore to implement a system of brain training games with NFT on a single channel using a consumer-grade EEG headset, and to evaluate its efficacy for cognitive training in elderly participants. Physiological evidence in terms of EEG was also investigated to determine whether the brain rhythms of interest change as expected after training.

## Methods

### System Overview

**Figure 1:**
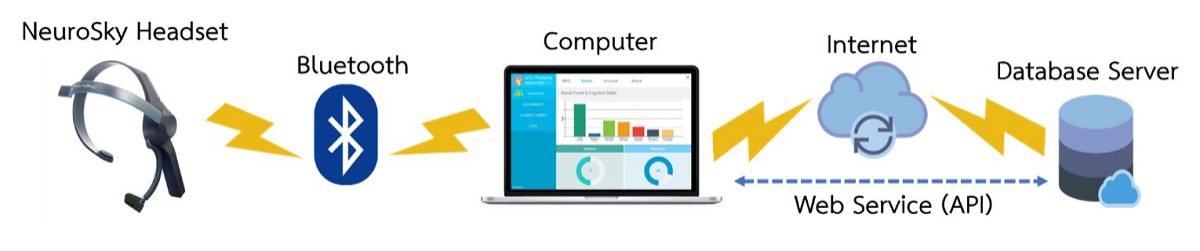
System overview. API: application programming interface.

Overall, the architecture of the NFT gaming system was designed to support multiuser application in multiple locations. At each location, the PC-based games ran locally on the computer, with the Neurosky EEG headset connected via Bluetooth to provide brain signal feedback. Training data were collected and sent to the remote database server over the internet (Figure 1).

**Figure 2:**
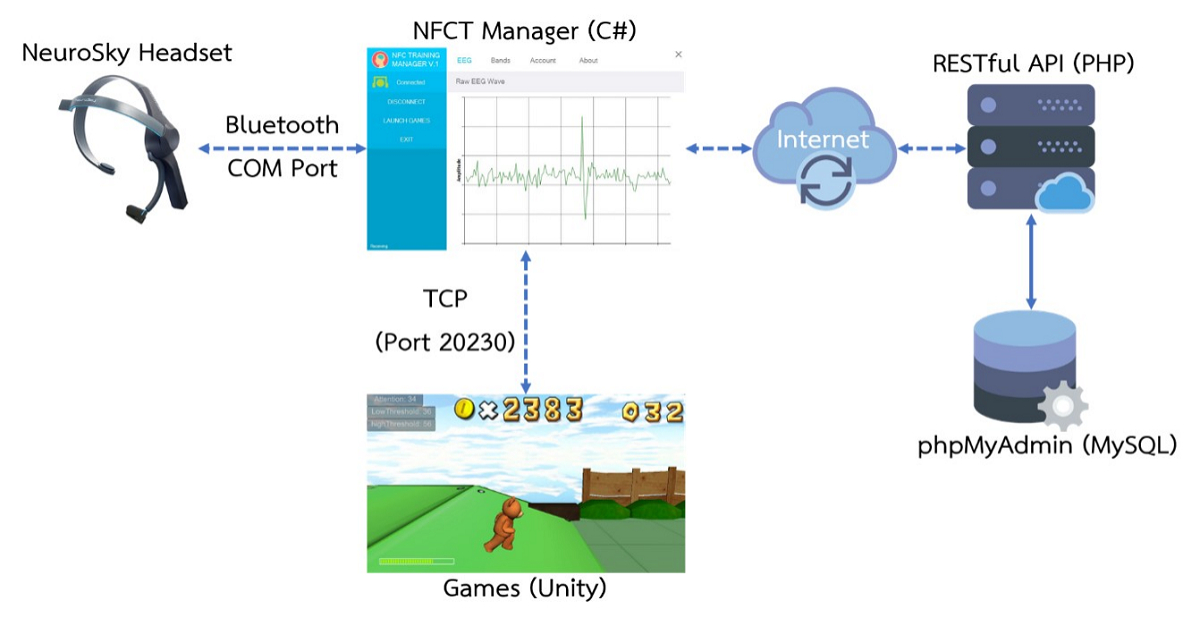
System configuration. NFCT: Neurofeedback Cognitive Training; API: application programming interface.

In terms of software, the games were developed using the Unity game development platform [54], and were managed by the Neurofeedback Cognitive Training Manager program written in C#. Training data were delivered to the server via the RESTful application programming interface (API) web service. The server was programmed using PHP with the MySQL database. Parameters such as EEG readings, scoring, and usage time/characteristics of each user could be collected for analysis via the system’s web service API (Figure 2).

### NFT Control and EEG Signal Processing

#### Selection of Feedback Signals

Different strategies for selecting which brain rhythms to be fed back for training had been considered, as discussed previously [45,55]. In general, there are two classical directions in NFT, focusing on either low frequencies (alpha or theta) to strengthen relaxation and focus or on high frequencies (low beta, beta, and theta) for reinforcing activation, organizing, and inhibiting distractibility. More specifically, the alpha protocol (ie, using the alpha wave as the feedback signal) has historically been used for relaxation training. Beta was considered to be associated with mental performance, and was used to improve focus or attention; alpha/theta has been used for stress reduction; gamma is a high-frequency band associated with cognitive processing and memory; and theta (including theta/beta) has been considered for anxiety, depression, ADHD, and emotional disorder treatments.

For this study, we decided on the type of feedback signals for our cognitive training scheme based on our observation that the strongest evidence in the cognitive training domain has been related to attention [26,29,30,53]. Attention was defined as the ability to focus cognitive resources on one relevant aspect of the environment while ignoring other irrelevant aspects. A significant portion of the published attention-based NFT literature suggests the use of alpha and beta rhythms [45,55-58]. For example, Li et al [58] provided evidence that the beta band (during a task) contained considerable information about attention, indicating the possibility of recognizing a subject’s attention level by studying the EEG data. In addition, according to Klimesch [36], the alpha band showed task-related desynchronization; that is, it increased during resting states (especially when the eyes were closed) and decreased during performance of a cognitive task (eg, mental calculations). Therefore, it was reasonable to try to mimic the phenomena observed by the good performers by means of NFT (ie, to enhance beta power and reduce alpha power during active mental tasks) to enhance cognitive performance. Accordingly, our system targeted training of attention as the first cognitive function for improvement. Correlations between attention and other cognitive functions such as memory have previously been discussed. For example, memory showed limited capacity, and thus attention determined what would be encoded [59]. We hypothesized that improvement in higher attention could also lead to improvement in other cognitive domains.

In our previous work [53], the feedback was defined as the attention level calculated as:


Original attention feedback = K_β_×P_β_/K_α_×P_α_

Where K_β_ and K_α_ are empirical scaling constants, P_β_ is the power spectral density (PSD) of the beta band (12-32 Hz), and P_α_ is the PSD of the alpha band (8-12 Hz).

With recent studies showing that theta (4-8 Hz) training that could affect memory performance [38], with beta low (12-16 Hz) specifically found to affect attention [60], we revised the attention feedback level as follows:


New attention feedback = (K_β_×P_βL_)/(K_αL_×P_αL_+K_θ_×P_θ_)

Where P_βL_ is the PSD of the beta low band (12-16 Hz), P_αL_ is the PSD of the alpha low band (8-10 Hz), and P_θ_ is the PSD of the theta band (4-8 Hz).

As the individual upper alpha (IUA) has been shown to affect cognitive power [38], the value of cognitive level monitored was then calculated from:

Cognitive level = (K_β_×P_βL_+K_αh_×P_αh_)/ (K_αL_×P_αL_+K_θ_×P_θ_)

Where P_αh_ is the PSD of the alpha high band (10-12 Hz).

#### Adaptive Threshold Control

**Figure 3:**
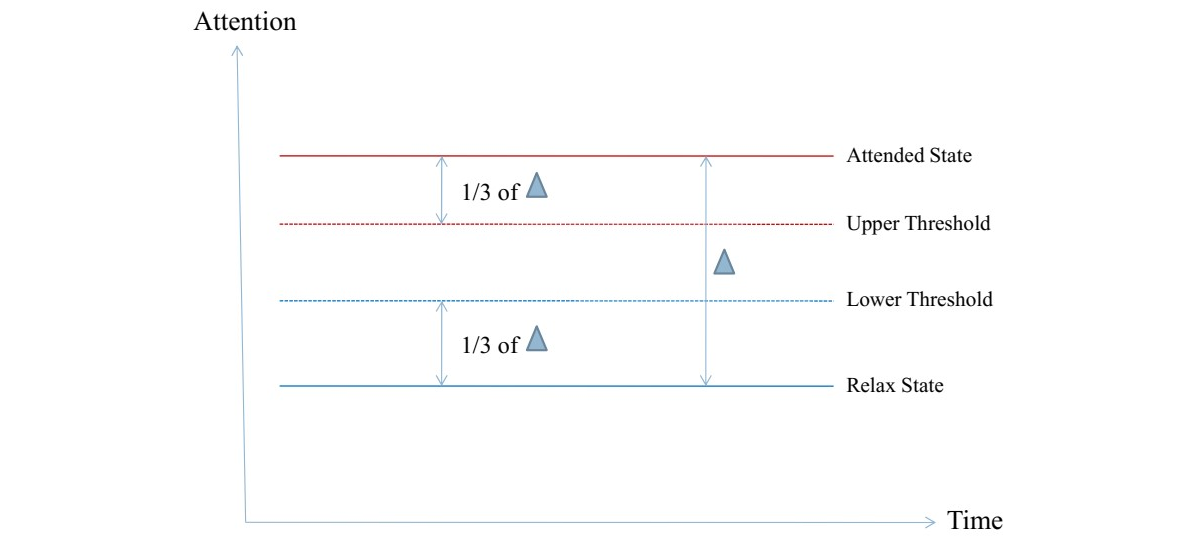
Setting upper and lower thresholds.

**Figure 4:**
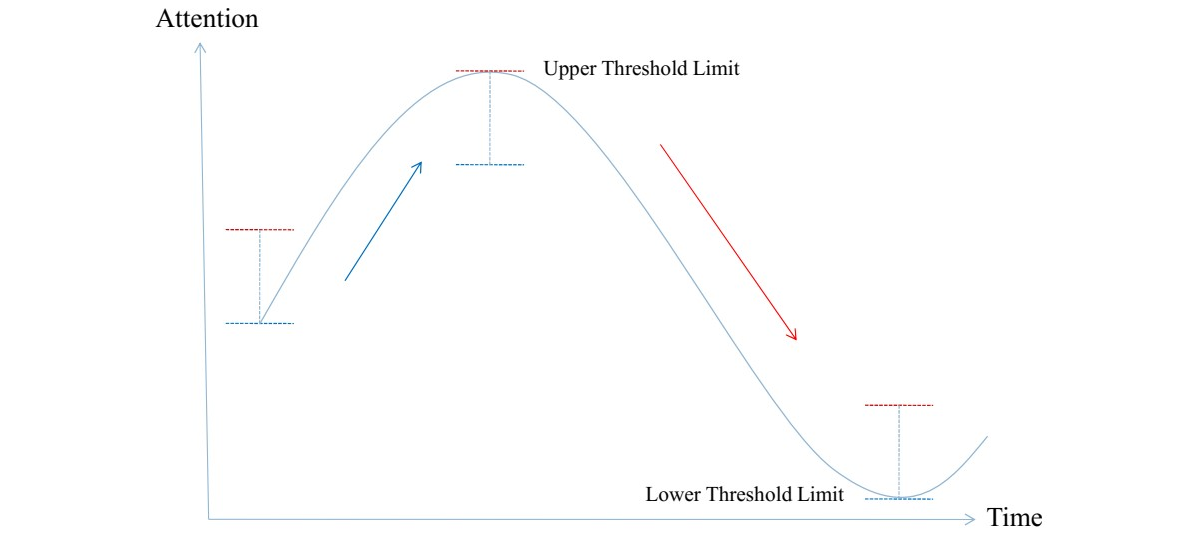
Adaptive threshold.

In our system, the brain feedback signal (attention) was used as a game-controlling signal in an adaptive manner, with two additional parameters introduced: the upper and lower thresholds. Before each session, the system would measure the user’s level of attention under two mental states: being attentive and relaxed. The difference between the two values was used to calculate the upper and lower threshold levels. Figure 3 shows an example of the condition when the lower and upper thresholds were adjusted by one third of the difference from the relaxed and attentive states, respectively. During the game play, if the user’s attention level was higher than the threshold level, they were rewarded such as by having the controlled character moving faster. By contrast, if the attention level was lower than the lower threshold, the character would slow down. Attention levels between the two thresholds had no effects on the game being played. As the brain signal is highly volatile and is known to exhibit session-to-session variation, the value of the thresholds would continuously adapt to suit the present situation (Figure 4). For example, if the user became fatigued and the attention level dropped down for too long, the thresholds would adapt so that the user could hit the upper threshold more easily. This was important so that the user could feel that the game was responsive and therefore retain their engagement.

### Game Description

**Figure 5:**
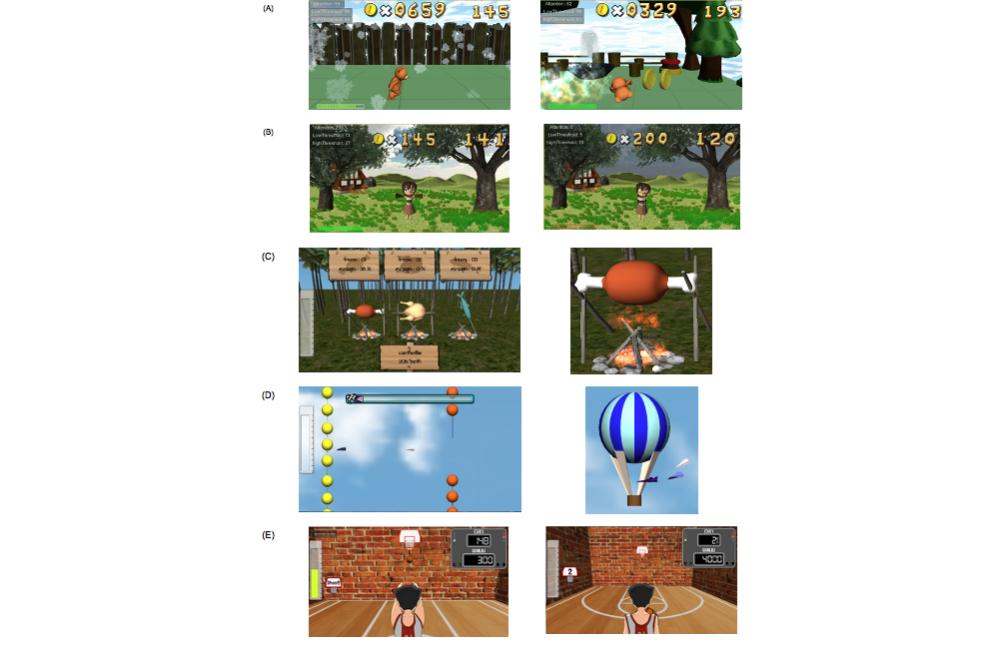
Screenshots of games: (A) Run Run, (B) Sunshine Day, (C) Cast Away, (D) Paper Plane, (E) 321 Shoot.

In terms of game design, there were five games in our NFT system (Figure 5). The main concept used for all 5 games was based on the same idea that the main character’s moving characteristic is controlled by the attention level feedback. The different scenarios between the games were mainly aimed at maintaining the user’s engagement. In “Run Run,” the first game, the bear runs faster when the player focuses on the game (ie, attention level above the upper threshold) and runs slower when the user loses focus (ie, attention below the lower threshold). The bear could also jump to collect coins if he ran fast enough, which was designed to provide the user more incentive to focus. For the second game, termed “Sunshine Day,” if the player focused on the girl character to cheer her up, the girl in the garden would look happy and the character would jump to indicate that she’s in a good mood. In “Cast Away,” the third game, the player’s attention level controls the cooking fire. There are three types of food, each of which requires a different level of cooking heat to be cooked. If the fire is strong, the food will be cooked quickly, resulting in high game scores. The fourth game was called “Paper Plane.” If the attention value is high, the plane moves fast and is able to fly away from obstacles. Competing against paper planes that are computer-controlled was also provided for motivation. The last game was “321 Shoot.” In this game, the average attention level was calculated every 3 seconds. If it was above the set threshold, then the basketball player scored. After every 10 points, the threshold was increased, and the basketball player would stand further away from the hoop.

### Trial Protocol

#### Design and Setting

The trial was designed as a multisite pilot study. Forty participants were targeted for the intervention by undergoing training at the following 5 participating hospitals and health centers in Thailand: King Narai Hospital; Queen Savang Vadhana Memorial Hospital; Thai Redcross Health Station 2, Sukuman Health Center; Thai Redcross Health Station 5, Sawangkanives; and Ban Bang Khae Social Welfare Development Center for Older Persons.

Intervention results were compared with those obtained from the control group, who received care-as-usual treatment.

#### Participants and Eligibility

Subjects were recruited on a voluntary basis from the participating sites. All subjects who had received a diagnosis of normal aging elderly and MCI were invited to participate. Diagnoses were made by psychiatrists according to standardized clinical criteria (Thai Mental State Examination [TMSE] and Montreal Cognitive Assessment [MoCA]-Thai) and the criteria for MCI established by Petersen et al [11].

The inclusion criteria were 55-80 years old, with adequate verbal expression, visual and hearing abilities; at least 4 years of education; ≥24 on the TMSE and ≤24 on the MoCA-Thai for MCI patients, or ≥24 on the TMSE and >24 on the MoCA-Thai for normal aging elderly; and not currently enrolled in another research study.

The exclusion criteria were presence or history of a confounding central neurologic disease (eg, brain tumor, stroke, epilepsy); currently under acetyl cholinesterase inhibitor treatment (eg, donepezil, galantamine, and rivastigmine); and presence of substance abuse disorder or substance dependence.

#### Experimental Protocol

At the pretest period, all test subjects were assessed for cognitive functions via Cambridge Neuropsychological Test Automated Battery (CANTAB) tests. The data, together with EEG records, were collected as baseline data. The intervention period started no later than 1 week after the assessment. The intervention group trained via NFT for attention for 30 minutes per session with 2 sessions per week for a period of 10 weeks (total 20 sessions). The subjects were then evaluated for cognitive functions outcome at week 10. Posttraining EEG data were also collected for analysis.

The study was approved by the Institutional Review Board of the Faculty of Medicine, Chulalongkorn University, Bangkok, Thailand (593/57). All participants provided written informed consent, and the study was performed in accordance with the Declaration of Helsinki.

### Cognitive Performance (CANTAB)

#### Delayed Matching to Sample

Delayed matching to sample (DMS) was used to assess both simultaneous visual matching ability and short-term visual recognition memory for nonverbalizable patterns. Each user was shown a complex visual pattern (abstract and nonverbal), followed by four similar patterns after a brief delay (0, 4, or 12 seconds). The pattern that exactly matched the sample had to be selected (Table 1).

**Table 1:** Parameters used for delayed matching to sample assessment.

Parameters	Meaning
%correct	Percentage of occasions upon which the subject selected the correct stimulus
%correct (simultaneous)	Percentage of occasions upon which the subject selected the correct stimulus in trials when the target stimulus and the three distractors were presented after the stimulus had been hidden, with delays of 0 ms
%correct (all delays)	The percentage of occasions upon which the subject selected the correct stimulus in trials when the target stimulus and the three distractors were presented after the stimulus had been hidden, with delays of 0 ms, 4000 ms, and 12,000 ms
Median correct latency	Median latency considering all occasions where the subject selected the correct stimulus on their first response in trials when the target stimulus and the three distractors were presented after the stimulus had been hidden after a specified delay. Latency was measured in milliseconds; a lower score was better
Prob error given error	Probability of an error occurring when the previous trial was responded to incorrectly

#### Motor Screening Task

The motor screening task (MOT) was used to assess whether sensorimotor deficits or lack of comprehension would limit the collection of valid data from the participant. For this test, colored crosses were presented in different locations on the screen, one at a time. The participant had to select the cross on the screen as quickly and accurately as possible (Table 2).

**Table 2:** Parameters in the motor screening task assessment.

Parameters	Meaning
Mean latency	Arithmetic mean of latency, defined as the time taken for the subject to touch the cross after it appeared. Latency was measured in milliseconds; a lower mean was better
Median latency	Arithmetic median of latency
Mean error	Measure of the subject’s pointing accuracy, according to the mean distance between the center of the cross and the location the subject touched on the screen, for the 10 crosses presented to which the subject correctly responded; a lower error was better

#### Pattern Recognition Memory

Pattern recognition memory was applied as a test of visual pattern recognition memory. The participant was presented with a series of visual patterns, one at a time, in the center of the screen. These patterns were designed so that they could not easily be given verbal labels. In the recognition phase, the participant was required to choose between a pattern they had already seen and a novel pattern in a 2-choice forced discrimination paradigm. In addition, the test patterns in this phase were presented in reverse order to the original order of presentation. This was then repeated, immediately or after a delay, with new patterns (Table 3).

**Table 3:** Parameters for the pattern recognition memory assessment.

Parameters	Meaning
%correct	Number of correct responses, expressed as a percentage
Mean correct latency	Arithmetic mean of the correct responses; a lower value was better
Median correct latency	Arithmetic median of the correct responses; a lower value was better
#correct	Number of correct answers given by the subject

#### Rapid Visual Information Processing

The rapid visual information processing (RVP) assessment was designed to measure sustained attention. At a rate of 100 digits per minute, digits from 2 to 9 appeared in a pseudorandom order inside a white box shown at the center of the screen. Participants were requested to detect target sequences of digits (eg, 3-5-6). The button in the center of the screen had to be selected as quickly as possible when the target sequence was noted. The level of difficulty varied with either one- or three-target sequences that the participant had to watch for at the same time (Table 4).

**Table 4:** Parameters for rapid visual information processing assessment.

Parameters	Meaning
A'	How effective the subject was at detecting target sequences
Probability of hit	The probability of the subject responding correctly; a higher value was better
Total false alarms	Number of times the subject responded outside the response window of a target sequence; a lower value was better
Mean latency	Mean time taken to respond (milliseconds)
Median latency	Measure of the median time taken to respond
Probability of false alarm	Probability of the subject responding inappropriately, equal to total false alarms/(total false alarms + total correct rejections); a lower value was better

#### Spatial Span

A spatial span test was used to assess visuospatial working memory capacity. White squares were shown on the screen, some of which briefly changed color in a variable sequence. The boxes that changed color in the same order in which they were displayed by the computer (for the forward variant) or in the reverse order (for the backward variant) had to be selected. The number of boxes in the sequence increased from two at the start of the test to nine at the end of the sequence, and the colors were varied throughout the test (Table 5).

**Table 5:** Parameters in the spatial span assessment.

Parameters	Meaning
Span length	The longest sequence successfully recalled by the subject
Mean time to first response (span length 3)	The time the subject took to initiate problems of the span length specified by the span length option (3). The time was measured from the end of the presentation phase (the moment the final box closed) until the subject touched the screen. Attempts undertaken on spans that the subject did not pass were included in this calculation. A lower value was better.
Total errors	Number of times the subject selected an incorrect box; a lower value was better
Mean time to last response (span length 3)	Time the subject took to complete problems of the span length (3). The time was measured from the end of the presentation phase (the moment the final box closes) to the time of the subject’s final response on a given attempt. Attempts undertaken on spans that the subject did not pass were included in this calculation. A lower value was better.

#### Spatial Working Memory

The spatial working memory (SWM) test required the retention and manipulation of visuospatial information, and provided a measure of strategy as well as working memory errors. First, several colored squares (boxes) were shown on the screen. By selecting the boxes and using a process of elimination, the participant had to find one yellow “token” in each of a given number of boxes and use them to fill up an empty column on the righthand side of the screen. The number of boxes could be gradually increased until a maximum of 12 boxes were shown for the participants to search. The color and position of the boxes used also changed from trial to trial to discourage the use of stereotyped search strategies (Table 6).

**Table 6:** Parameters for spatial working memory (SWM) assessment.

Parameters	Meaning
Between errors	Results for trials containing the number of boxes specified by n (4, 6, or 8) only; a lower value was better
Strategy	Specifies the lower and upper numbers of boxes to calculate the SWM strategy measure; a lower value was better
Median time to first response	Mean time between the problem being presented to the subject and the subject first touching the screen to open a box, for problems with the specified number of boxes; a lower value was better
Median time to last response	Mean time of the subject’s last response for a problem, calculated from the time between the problem being presented to the subject and the subject’s last screen touch to open a box to locate the final token for the problem; a lower value was better

### Physiological Brain Characteristics

Before the first training session, each participant recoded their brain EEG signals using a research-grade multichannel g.Nautilus headset from g.tec medical engineering. The g.Nautilus device is a wireless EEG sensing and amplification system with a 24-bit resolution at a 250/500 Hz sampling rate. The version used was configured to work with g.SAHARA hybrid EEG electrodes for dry or wet recordings. After asking the participant to relax, the EEG signals were recorded at 16 positions (Fp1, Fp2, F3, Fz, F4, T7, C3, Cz, C4, T8, P2, Pz, P4, PO7, PO8, and Pz) when the eyes were both open and closed. The entire procedure was repeated after the last training session (session 20). 

## Results

### Participants

A total of 104 individuals applied for the intervention program. Seventeen were excluded from the study based on their health conditions. A further 22 candidates did not meet the TMSE and MoCA criteria. Of the remaining candidates, 43 individuals eventually participated in the program and 35 completed the training (8 dropouts).

### CANTAB Results

Table 7 shows the baseline CANTAB values of the control and intervention groups. Analysis results of CANTAB readings between pre- and posttraining using paired *t* tests are shown in Table 8.

Statistically significant improvements were found in terms of DMS (%correct, %correct all delays), median latency, and SWM (between errors), meaning that there were benefits from the training in terms of visual memory (DMS), attention (MOT), and visual recognition (SWM). Improvements were also found in the control group in terms of RVP (probability of hit, probability of false alarm) and MOT (median latency, mean error).

The results indicated that after brain exercising based on the proposed NFT system, we did find improvements in both memory and attention domains as we had hoped. DMS was the test of visual matching to sample, assessing mainly visual memory, and depended heavily on a forced-choice decision that may be dependent on frontal as well as temporal lobe functions. By contrast, the SWM score depended on the ability to retain spatial information to manipulate remembered items in working memory. Therefore, the improved SWM score could indicate improvements in the frontal lobe as well as in executive function.

**Table 7:** Baseline information of the participants.

Characteristic	Control (n=30)	Neurofeedback training (n=35)
Age (years), mean (SD)	69.86 (8.95)	69.48 (6.15)
Female, n (%)	28 (93)	31 (89)
TSME^a^, mean (SD)	27.50 (1.70)	27.09 (2.05)
MoCA^b^, mean (SD)	20.83 (2.53)	21.00 (2.03)

^a^TMSE: Thai Mental State Examination.

^b^MoCA: Montreal Cognitive Assessment.

**Table 8:** Cambridge Neuropsychological Test Automated Battery results.

Parameter^a^		Control				Neurofeedback training		
		*t* (*df*=29)	SD (x – y)	*P* value	*t* (*df*=34)		SD (x – y)	*P* value
**DMS^b^**								
	%correct	–0.5177	8.8165	.61	–3.0566		9.2627	.004
	%correct (simultaneous)	1.2289	10.4	.23	–1.5082		14.5695	.14
	%correct (all delays)	–0.9581	11.4336	.35	–2.6199		11.6131	.01
	Median correct latency	–1.9927	1.02E+03	.06	0.3146		1.18E+03	.76
	Prob error given error	–0.4167	0.2088	.68	0.7981		0.1566	.43
**MOT^c^**								
	Mean latency	1.8352	244.0492	.08	1.0251		275.8327	.31
	Median latency	3.6956	166.6858	<.001	2.7713		213.8717	.009
	Mean error	–2.5181	2.7958	.02	–0.9254		5.1444	.36
**PRM^d^**								
	%correct	–0.1606	9.472	.87	–1.1758		14.9745	.25
	Mean correct latency	–0.5861	596.2864	.56	0.8479		1.18E+03	.40
	Median correct latency	–1.6612	248.2209	.11	1.4163		502.0288	.17
	#correct	–0.1575	2.3183	.88	–1.1758		3.5939	.25
**RVP^e^**								
	A'	–2.041	0.0535	.05	–0.3157		0.0802	.75
	Probability of hit	–2.3338	0.1766	.03	0.5545		0.1806	.58
	Total false alarms	0.3692	1.978	.71	0.8318		7.5184	.41
	Mean latency	–0.1099	90.2135	.91	1.6845		265.2661	.10
	Median latency	0.2864	110.6188	.78	1.9901		289.9302	.05
	Probability of false alarm	2.2401	0.0083	.03	0.8618		0.0325	.39
**SSP^f^**								
	Span length	1.27	1.0063	.21	–1.0964		1.0792	.28
	Mean time to first response (span length 3)	–0.9127	622.248	.37	1.2085		1.50E+03	.24
	Total errors	0.1436	6.3576	.89	0.2642		7.0368	.79
	Mean time to last response (span length 3)	1.5412	1.32E+03	.13	1.0786		2.43E+03	.29
**SWM^g^**								
	Between errors	–0.2158	9.3051	.83	2.2582		12.2757	.03
	Strategy	0.5781	2.8424	.57	0.0838		4.0362	.93
	Median time to first response	–1.5109	654.9549	.14	0.7888		991.3451	.44
	Median time to last response	–0.6447	5.89E+03	.52	1.1679		7.14E+03	.25

^a^See Tables 1-6 for a description of the parameters.

^b^DMS: delayed matching to sample.

^c^MOT: motor screening.

^d^PRM: pattern recognition memory.

^e^RVP: rapid visual information processing.

^f^SSP: spatial span.

^g^SWM: spatial working memory.

### EEG Results

**Figure 6:**
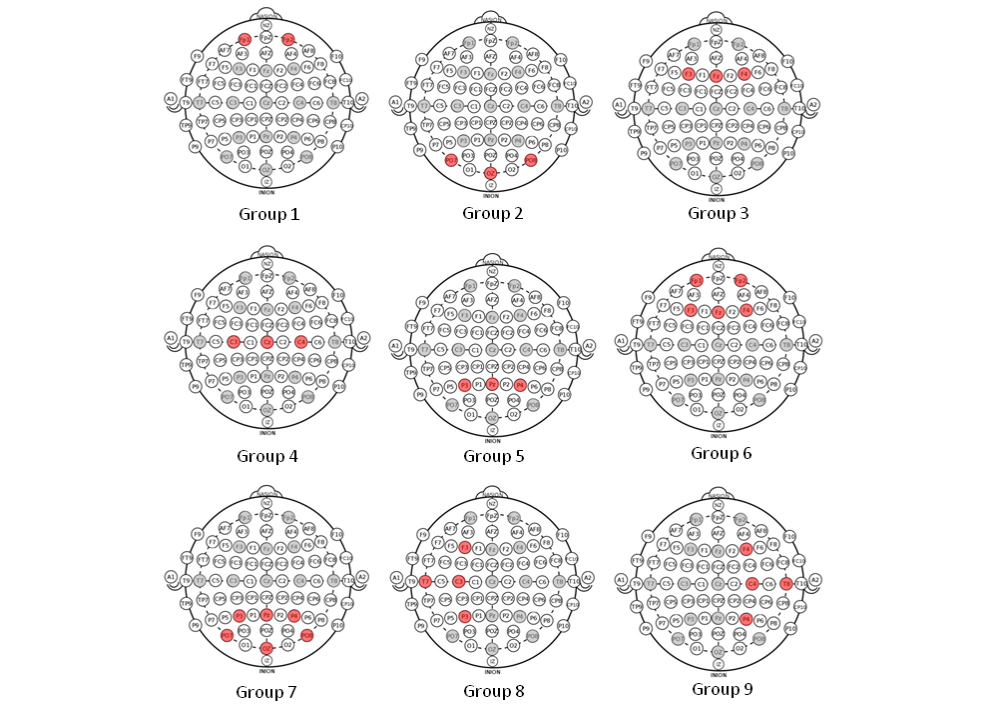
Groupings of electroencephalogram channels.

To analyze the EEG data, the channels were first grouped according to their cortical areas, as shown in Figure 6. This was necessary because of the known associations between brain patterns and where they would occur in the brain areas: the prefrontal (Group 1), occipital (Group 2), frontal (Group 3), central (Group 4), and parietal (Group 5) areas. Signals from the prefrontal and frontal areas (Group 6) and from the parietal and occipital areas (Group 7) were also analyzed together, respectively. EEGs from the temporal left and right sides were grouped as Group 8 and Group 9, respectively.

The PSD of each group was then calculated as the average of each channel’s PSD within the group, with the paired *t* test calculated from pretraining and posttraining group PSD values. Significant changes in alpha high were found during the open-eyed relaxed condition in Group 2 and Group 7 (*P*=.04 for both). This agreed well with the finding of Klimesch [38] that an increase in alpha high corresponded to improvement in cognitive performance. This observation in Group 2 and Group 7, which covered the occipital areas, further supported the validity of the results as these are the areas where alpha was expected to be more evident.

## Discussion

### Principal Results

The goal of this study was to investigate whether brain training games with consumer-grade single-channel EEG sensor neurofeedback could be effective in enhancing the cognitive functions of elderly users. The results seemed to confirm its usefulness, especially in terms of memory improvements in both DMS and SWM. Compared to our prior works, SWM improvement was indeed also observed in NFT in children [30] and in our previous trial with older subjects [53]. The difference in terms of the setup between this trial and the previous trial [53] was that this trial used single-channel EEG instead of multichannel EEG (Emotiv Epoc). A single-channel EEG was more practical and easier to administer, but signal reading quality could suffer as a result. Another main difference is that this trial also employed the new feedback signal and adaptive threshold concepts. All of these factors could have contributed to the slightly different results obtained between the two studies.

For the attention domain, a significant change was found on MOT compared to significant changes found for RVP in previous studies [30,53]. This difference could also be due to the difference in population and setup. Notably, the MOT change was also found in the control group. As MOT was designed primarily for screening purposes, we could speculate that it might have a sensitivity that made it less suitable for attention assessment.

In general, the results of this study are in line with the current literature given a similar focus on two main areas of potential benefits: attention [61-63] and working memory [64,65]. Evidently, the published results to date are variable. For example, Gadea et al [66] reported no significant changes on theta downregulation but achieved significant changes in attention. By contrast, other studies [67-69] reported successful voluntary modulation of brain activity (change in the EEG signal) but no effects on the behavior (eg, symptom reduction). Both effects were shown in other studies [70-74]. A recent study concerning NFT using low-cost EEG found alpha enhancement but no effect on cognitive performance [75]. There are many factors that may have contributed to these mixed results. Experimental variables (factors) such as protocol, duration of the training, location of electrodes, or signal modality could have been set up differently, leading to these mixed results. Indeed, the large majority of neurofeedback studies appeared to have at least one major methodological limitation such as lack of randomization, nonblind designs, and use of waiting list control conditions. Controls without sham groups in a quasiexperimental design used in many trials means that evidence for the efficacy of the independent variable (ie, feedback training) could not be fully validated. Unfortunately, use of a sham control for the positive effects is ethically challenging, since the use of placebo (which also includes sham groups) instead of clinical treatments may lead to a deterioration of symptoms. Thus, the well-controlled EEG-based neurofeedback studies performed to date could only be carried out on treatment-resistant or healthy subjects. In nonblind designs, factors such as the relationship with the therapist are also relevant, especially when treating children.

Another issue regarding the effectiveness of NFT is sustainability validated through long-term studies. There have only been a few such studies, mostly targeted toward ADHD treatment. For example Lee et al [62] and Rijken et al [76] evaluated the long-term effect of NFT through follow-up. Compared to nonactive control treatments, NFT appeared to have more durable treatment effects for at least 6 months following treatment [77]. The reasons for the lack of such studies may be related to practical issues such as difficulty to reconcile schedules with the participants or withdrawal of subjects.

Despite these limitations, it is still evident from literature reviews that there is a clear scientific movement toward the use of neurofeedback as a tool to improve cognition and behavior besides rehabilitation. Moreover, it is interesting to note a shift in the direction of research in this field, characterized by a move from the classical clinical standard of evaluating the effectiveness of the method (standardized double-blind, randomized experiment) to the use of other assessment methods that seem more appropriate for neuropsychological treatments. The primary reason is related to contradictions between the positive outcomes of single case studies and the ineffectiveness of studies with large numbers of subjects [78]. This trend eventually may lead to the development of individualized treatment protocols, where effect of the treatment is assessed within the single case.

With respect to the details of the design, for simplicity, we used alpha high with a fixed frequency range, given recent studies indicating that it was possible to enhance cognitive capacities of healthy individuals by means of IUA NFT, where the IUA was located between the individual alpha peak (IAP; between 7.5 and 12.5 Hz) and IAP+2 Hz [38]. Changes from upper alpha to IUA could have potential to improve the effectiveness further.

The training time of 30 minutes per session provided a user with considerable training time, while being short enough to not cause too much fatigue. During game development and testing, we found that an individual game duration of 2-3 minutes was appropriate. Game players usually spent the first 30 seconds up to 1 minute to ramp up their attention levels. These levels were then maintained or slightly improved throughout the game. After 2 minutes, the level of attention tended to drop, and several players felt fatigue and lost attention completely after 3 minutes. As a result, all 5 games were programmed to run for 2-3 minutes. Players were also asked to take a break after every game.

Each NFT set comprised a low-cost one-channel EEG headset and a PC with internet and Bluetooth connection. Once set up, the user could simply put on the headset and start a game. This could be a low-cost addition to community centers for the elderly around the country. These NFT games could be played leisurely to supplement existing activities such as group music and craft works.

Finally, as Staufenbiel et al [79] and Wang and Hsieh [80] pointed out, the literature remains sparse in relation to assessing the impact of neurofeedback in the elderly population. Our work, showing positive benefits on both EEG and cognitive functions, hopefully contributes as an additional step toward the confirmation of benefits for NFT in the elderly.

### Limitations

This study has some limitations that warrant future research. First, the sample size could be considered to be small. According to Begemann et al [81], to detect a medium effect size of 0.5, a minimal sample size of 64 per group would be needed. This criterion was not met by our study or most of the related works reported to date. Underpowered studies could carry the risk of both false-positive and false-negative findings, and are potentially more likely to be affected by publication bias, selective data analysis, and selective reporting of outcomes [82]. Moreover, as in the majority of similar research, our study design was quasiexperimental. This could also raise a question of how much the success observed in the modulation of the brain activity or behavior was due to actual training as opposed to nonspecific factors that could significantly contribute to the results. Although we have taken precautionary measures to limit these potential biases, a full randomized controlled trial with a higher number of samples would be one way to further solidify the findings. A long-term study would also be beneficial. To increase the chance for successful completion, the study could be designed with a shorter but more intensive intervention to encompass the follow-up phase with fewer possible withdrawals.

### Conclusions

In this work, we have demonstrated the potential benefit of game-based brain exercise using neurofeedback. The system implemented was based on a practical single-channel EEG using new feedback and gaming techniques. Results from the 5-site pilot study have demonstrated improvement in the visual memory (DMS), attention (MOT), and visual recognition (SWM) domains. EEG analyses also showed improvement in upper alpha at a resting state (open-eyed) in the frontal area, which similarly indicated improvement in the cognitive domain. 

## Abbreviations

ADHD: attention deficit hyperactivity disorder
API: application programming interface
CANTAB: Cambridge Neuropsychological Test Automated Battery
DMS: delayed matching to sample
EEG: electroencephalogram
IAP: individual alpha peak
IUA: individual upper alpha
MCI: mild cognitive impairment
MoCA: The Montreal Cognitive Assessment
MOT: motor screening task
NFT: neurofeedback training
PSD: power spectral density
RVP: rapid visual information processing
SWM: spatial working memory
TMSE: Thai Mental State Examination
